# Minimally Invasive Plate Osteosynthesis (MIPO) for Proximal and Distal Fractures of The Tibia: A Biological Approach

**DOI:** 10.5704/MOJ.1603.006

**Published:** 2016-03

**Authors:** P Gupta, A Tiwari, A Thora, JK Gandhi, VP Jog

**Affiliations:** Department of Orthopaedics, Maulana Azad Medical College, New Delhi, India

**Keywords:** Biological fixation, indirect reduction, MIPO, tibialfracturesdamage

## Abstract

**Introduction:** The treatment of fractures of proximal and distal tibia is challenging, because of the limited soft tissue envelope and poor vascularity. The best treatment remains controversial and it depends on the fracture morphology, displacement and comminution. Treatment options vary from closed reduction and cast to open reduction and internal fixation with plate. Open reduction and internal fixation with plate can result in extensive dissection and tissue devitalization. We conducted a study on management of these fractures by biological osteosynthesis using Minimally Invasive Plate Osteosynthesis (MIPO) technique with preservation of osseous and soft tissue vascularity.

**Methods:** We conducted a prospective study on closed reduction and percutaneous plating in 30 cases (mean age 42.7 years; 22 males and 8 females) of closed fractures of tibia. Among them 24 had proximal tibial fractures and 6 had distal tibial fractures. The mean time from injury to surgery was seven days.

**Results:** The mean operative time was 72.6 minutes ( range: 55-90 minutes). Mean time for radiological union was 17 weeks (range: 14-22 weeks). There was one superficial wound infection which resolved with daily dressings and one week of oral antibiotics. One patient developed a nonunion which required a bone grafting procedure.

**Conclusions:** The satisfactory functional results and lack of soft tissue complications suggest that this method should be considered in periarticular fractures. Biological fixation of complex fractures gives stable as well as optimal internal fixation and complete recovery of limb function at an early stage with minimal risk of complications.

## Introduction

The goal of proximal and distal tibial fracture treatment is to obtain early union of fracture in the most acceptable anatomical position with early and maximum functional return of activity. In view of the ever increasing high velocity road traffic accidents, there is increase in complex, multifragmentary periarticular fractures of the tibia. Treatment modalities of fractures of tibia are closed reduction and cast application, closed reduction and external fixation, closed reduction and internal fixation with Minimally Invasive Plate Osteosynthesis (MIPO) technique and open reduction and internal fixation with plate. Each method has its own advantages and disadvantages.

Non-operative treatment of closed comminuted fractures with cast usually leads to problems like prolonged immobilization, malunion, shortening and joint stiffness. Open reduction and internal fixation with conventional plate frequently lead to complications like non-union, delayed union, infection and implant failure. The single most important factor in the treatment of these fractures is the management of overlying soft tissues. Rhinelander^1^ (1968) believed that blood supply is the most important factor in normal bone healing. So while using the technique of internal fixation, emphasis must be on the vascular support of bone and soft tissue by doing minimum exposure, indirect reduction and in particular the least possible damage to periosteum.

Therefore, the concept of management of these fractures has been changed from absolute fixation to relative fixation of biological osteosynthesis with preservation of osseous and soft tissue vascularity. Biological plating provides relative stability and preserves vascularity around the fractures. The principles of this minimally invasive technique include indirect closed reduction, extraperiosteal dissection and relative stability which allows limited controlled motion at the fracture site with secondary bone healing with callus formation^2^. The present prospective study was to evaluate the efficacy of MIPO technique in the management of closed proximal and distal fractures of the tibia.

## Materials and Methods

**Fig. 1:**
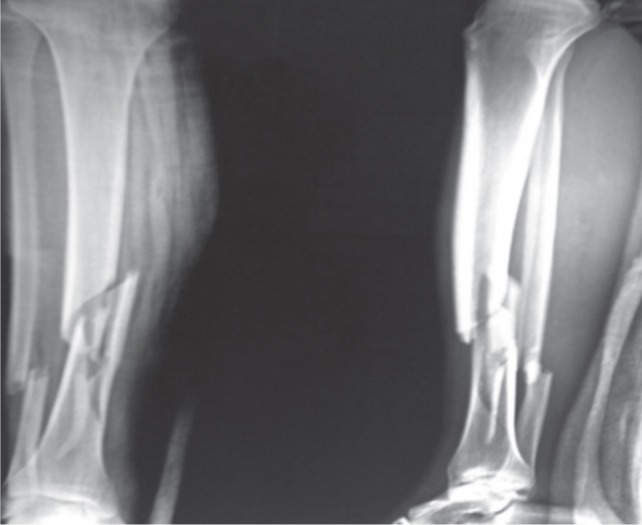
Radiograph showing the fracture of tibia and fibula at junction of proximal two-third and distal one-third.

This prospective study was conducted from April 2011 to June 2013. In this study 30 patients with acute-closed fractures of tibia were included. Among them 24 had proximal tibial fractures and six -distal tibial fractures. Open fractures, fractures with neurovascular injury, and pathological fractures were excluded from the study. Fractures were classified using AO classification, Type A (n=14): Type B (n=8): Type C (n=8). Patients selected for the study underwent pre-anaesthetic checkup and radiographs of the affected limb in anteroposterior and lateral views (Figure 1). After written informed consent, the patients were operated under spinal anaesthesia. The tourniquet was applied in the upper thigh. Tibia was exposed proximal and distal to the fracture site, fracture reduction was achieved by indirect reduction techniques with the help of pointed reduction forceps, external fixator, articular tensioning device or bone spreaders.

**Fig. 2:**
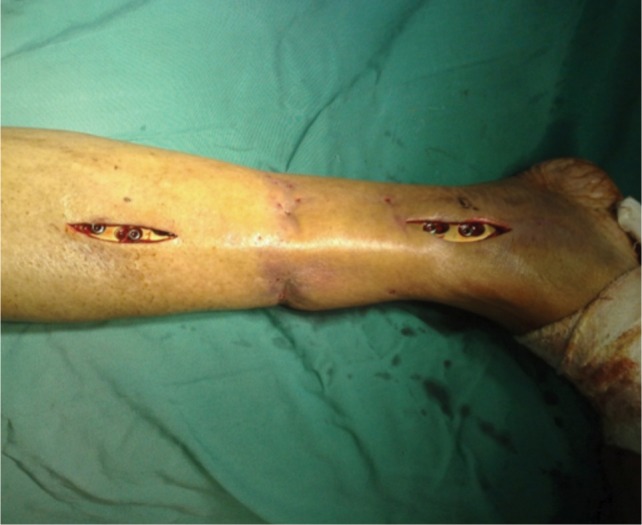
Clinical photograph showing the two mini incisions and fixation with a locking plate.

**Fig. 3:**
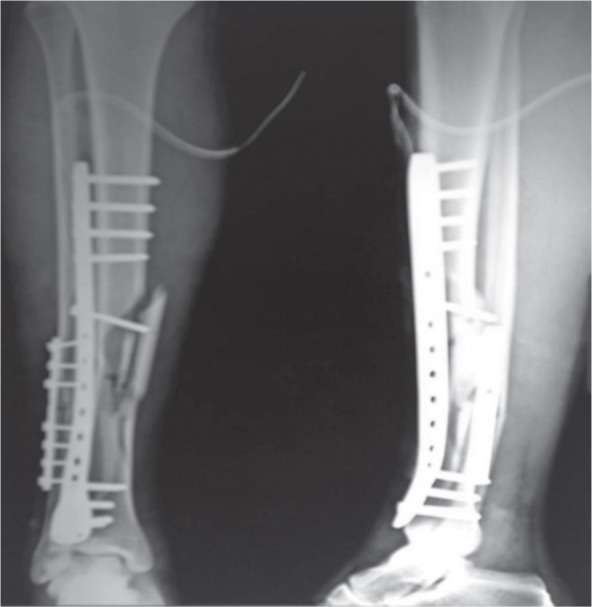
Anteroposterior and Lateral radiograph showing fracture fixation of tibia and fibula of same patient shown in Figure 2.

A tunnel was made submuscularly with the help of Cobb’s elevator. The plate was passed through this tunnel with the help of thread tied to one end and pulled with the help of a rongeur and fixed with screws on either side under fluoroscopic guidance (Figure 2). Each fragment was fixed on either side with a purchase of minimum six cortices. Wound was closed in layers. All patients received a single dose antibiotics preoperatively and post-operatively for 24 hours. All patients were given posterior above knee splint which was removed on the second post-operative day. Static quadriceps exercises and knee and ankle range of movement exercises were started the day following surgery. Postoperative radiographs were done on the day following surgery (Figure 3).

Non-weight bearing ambulation was started on the second post-operative day. Wound was inspected on the second postoperative day and sutures were removed on the 12th postoperative day. Partial weight bearing ambulation was started from six weeks and full weight bearing after 12 weeks when sufficient callus was seen on radiograph. On an average, all the patients were able to bear full weight on the operated limb from 12 weeks onwards, except the one case with delayed union which ultimately united at 22 weeks after bone grafting after which full weight bearing was allowed. Patients were assessed for pain at fracture site, tenderness, range of movement at knee and ankle, operative scar and radiological union at 6, 10, 14, 18, 22 weeks, 6 months and 12 months.

## Results

**Fig. 4:**
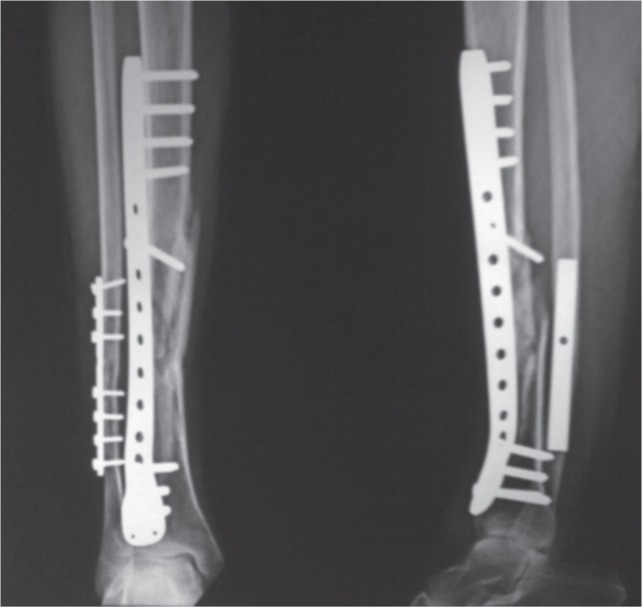
Anteroposterior and Lateral radiograph showing union of fracture of tibia and fibula of same patient shown in Figure 2 and 3.

In our study, 30 patients of proximal and distal tibial fractures were treated with closed reduction and internal fixation with MIPO technique. There were 22 males and 8 females, age range from 18 to 70 years with a mean age of 42.7 years. Left tibial fracture was in 13 cases and right in 17 cases; proximal tibial fracture was in 24 cases and distal tibial fractures in six cases; 20 cases were caused by road traffic accident, five cases of domestic fall, and five cases of physical assault. Majority of the patients were operated within the first week of injury (60%), mean time from trauma to surgery was seven days. The mean operative time was 72.6 minutes (55-90 minutes). Mean time for radiological union was 17 weeks (14-22 weeks) (Figure 4). All patients were followed up for minimum of one year postoperatively.

There was one superficial wound infection which resolved with daily dressings and one week of oral antibiotics. One patient non-union for which autogenous bone grafting from illiac crest was done at 12 weeks and the fracture was united at 22 weeks.

Bony and functional results were classified into four categories ranging from excellent to poor according to SJLAM criteria (1964)^3^ :

**Table:** 

Excellent:	Range of movement of adjacent joints 80-100 % of normal. No pain in performing daily activities. Good: Range of movement of adjacent joints 60 -80% normal. Pain not enough to cause any modification of patient daily routine.
Fair:	Range of movement of adjacent joints 30–60% normal. Pain enough to cause restriction patients daily activities.
Poor:	Range of movement of adjacent joints less than 30% of normal. Pain enough to cause severe disability or non union.

In our study 18 patients (60%) had excellent results, 10 patients (33%) had good results and 2 patients (7%) had fair result.

## Discussion

The management of proximal and distal fractures of the tibia requires individualized decision making. Non-operative treatment is best for stable fractures with minimal shortening, but malunion, shortening, stiffness and osteoarthritis of adjacent joint have all been reported following treatment of these fractures^4,5^. Open reduction of distal tibia fractures and internal fixation with plate require a large incision, extensive soft tissue dissection and periosteal stripping for anatomical reduction with complications including infection (range 8.3%–23%)^6,7^ delayed union and non-union (range 8.3%–35%)^8,9,10^. The surgical dissection required for achieving anatomical reduction causes soft tissue stripping and drains the fracture haematoma resulting in infection, delayed union and non-union^11^. A balance between anatomical reduction and soft tissue stripping is required in order to avoid these complications.

**Table I:** Recent (within 5 years) clinical studies (minimum 10 patients) utilizing stem cell use in orthopaedic surgery, sorte d by levels of evidence

S. No	Age (years)	Gender	Injury to Surgery time (Days)	Mode of Trauma	Type of fracture	Implants	Post op 6 weeks	Post op 12 weeks	Final follow up	Time to Complications union (weeks)	
1	40	M	10	RTA	AO B.1.2	a) hockey plate 9 hole b) 3 CCS	a) no pain b) knee rom 80 c) xray - no callus seen	a) no pain b) knee rom - 90 c) xray -not uniting	a) no pain b) knee rom 90 c) xray not uniting	-	Non union
2	40	M	7	Domestic fall	AO B.1.2	a) Hockey plate 7 hole b) 3 CCS	a) no pain b) knee rom 90 c) xray – uniting	a) no pain b) knee rom - 100 c) xray – uniting	a) no pain b) knee rom 110 c) full wt bearing d) xray united	20	No
3	29	M	2	Assault	AO C .1.1	a) hockey plate 11 hole b) 3 CCS	a) no pain b) knee rom 70 c) xray – uniting	a) no pain b) knee rom - 90 c) xray – uniting	a) no pain b) knee rom 100 c) full wt bearing d) xray united	15	No
4	40	M	8	Assault	AO B.1.2	a) hockey plate 7 hole b) 3 CCS	a) no pain b) knee rom -100 c) xray – uniting	a) no pain b) knee rom - 110 c) xray - uniting	a) no pain b) knee rom 120 c) full wt bearing d) xray united	16	No
5	17	M	6	RTA	AO B.1.3	a)13 hole Distal tibia locking plate	a) pain at fracture site b) ankle rom -5(DF)/10(PF) c) xray – uniting	a) no pain b) ankle rom -10(DF)/20(PF) c) xray - uniting	a) no pain b) ankle rom-10(DF)/20(PF) c) full wt bearing d) xray united	22	Superficial infection
6	65	F	6	Domestic fall	AO A.1.1	a) hockey plate 9 hole	a) no pain b) knee rom -80 c) xray – uniting	a) no pain b) knee rom - 80 c) xray - uniting	a) no pain b) knee rom 90 c) full wt bearing d) xray united	19	No
7	52	M	6	RTA	AO A 1.3	a) Recon LCP 9 hole b) 3 locking screws	a) no pain b) knee rom - 90 c) ankle rom full d) xray – uniting	a) no pain b) knee rom - 100 c) ankle rom full d) xray – uniting	a) no pain b) knee rom 110 c) full wt bearing d) xray united	16	No
8	60	M	9	RTA	AO C 1.3	a) Distal tibia locking plate 7 hole b) 6 locking screws	a) no pain b) ankle rom 10(DF)/ 20(PF) c) xray – uniting	a) no pain b) ankle rom – 10(DF)/30(PF) c) xray - uniting	a) no pain b) ankle rom 20(DF)/40(PF) c) full wt bearing d) xray united	15	No
9	60	F	9	Domestic fall	AO A 1.2	a) hockey plate 9 hole b) 3 CCS	a) no pain b)knee rom 50 c) xray – uniting	a) no pain b) knee rom - 80 c) xray - uniting	a) no pain b) knee rom 90 c) full wt bearing d) xray united	20	No
10	28	M	2	Assault	AO B 1.1	a) hockey plate 11 hole b) 3 CCS	a) no pain b) knee rom 90 c) xray - uniting	a) no pain b) knee rom - 100 c) xray – uniting	a) no pain b) knee rom 110 c) full wt bearing d) xray united	14	No
11	47	F	2	Domestic fall	AO A 2.1	a) Recon LCP 9 hole b) 6 Locking screws c) 1 IFS	a) no pain b) knee rom - 80 c) ankle rom full	a) no pain b) knee rom - 100 c) xray - uniting	a) no pain b) knee rom - 100 c) full wt bearing d) xray - uniting	18.5	No
12	58	M	6	RTA	AO A 1.3	a) Locking compression plate 7 hole b) 6 locking screws	a) no pain b) knee rom 70 c) xray - uniting	a) no pain b) knee rom - 80 c) xray - uniting	a) no pain b) knee rom 90 c) full wt bearing d) xray united	16	No
13	37	M	13	RTA	AO A 1.1	a) locking hockey plate 7 hole b) 6 locking screws	a) no pain b) knee rom 100 c) xray – uniting	a) no pain b) knee rom - 110 c) xray - uniting	a) no pain b) knee rom 120 c) full wt bearing d) xray united	19	No
14	38	M	10	RTA	AO A 1.3	a) locking hockey plate 9 hole b) 7 locking screws c) 1 IFS	a) no pain b) knee rom 90 c) xray - uniting	a) no painb) knee rom - 100c) xray – uniting	a) no pain b) knee rom 110 c) full wt bearing d) xray united	15	No
15	28	F	14	RTA	AO A 1.2	a) DCP 7 hole	a) no pain b) knee rom 80 c) xray – uniting	a) no pain b) knee rom - 90 c) xray - uniting	a) no pain b) knee rom 100 c) full wt bearing d) xray united	14	No
16	65	M	6	RTA	AO C 1.2	a) LCP 7 holeb) 6 locking screws	a) no pain b) knee rom – 50 c) xray - uniting	a) no pain b) knee rom - 60 c) xray – uniting	a) no pain b) knee rom 80 c) full wt bearing d) xray united	16	No
17	60	M	5	RTA	AO C 1.1	a) Distal tibia locking plate 7 hole b) 6 locking screws	a) no pain b) knee rom 80 c) xray – uniting	a) no pain b) knee rom - 90 c) xray - uniting	a) no pain b) knee rom 100 c) full wt bearing d) xray united	18	No
18	39	M	9	Assault	AO A 1.2	a) hockey plate 9 hole b) 4 CCS	a) no pain b) knee rom 100 c) xray – uniting	a) no pain b) knee rom - 110 c) xray – uniting	a) no pain b) knee rom 120 c) full wt bearing d) xray united	18	No
19	70	M	19	RTA	AO A 1.3	a) Locking hockey plate 9 hole	a) no pain b) knee rom 80 c) xray - uniting	a) no pain b) knee rom - 90 c) xray – uniting	a) no pain b) knee rom 90 c) full wt bearing d) xray united	20	No
20	26	M	3	RTA	AO C 1.3	a) Distal tibia locking plate 9 hole b) 7 locking screws	a) no pain b) ankle rom 15(DF)/30(PF) c) xray - uniting	a) no pain b) ankle rom 20(DF)/40(PF) c) xray - uniting	a) no pain b) ankle rom 20(DF)/ 40(PF) c) full wt bearing d) xray united	18	No
21	34	F	1	RTA	AO C 1.2	a) Distal tibia locking plate 9 hole b) 6 locking screws	a) no pain b) ankle rom 15(DF)/ 30(PF) c) xray - uniting	a) no pain b) ankle rom 15(DF)/30(PF) c) xray - uniting	a) no pain b) ankle rom 15(DF)/30(PF) c) full wt bearing d) xray united	19	No
22	35	M	3	RTA	AO B.1.2	a) Hockey plate 7 hole b) 3 ccs	a) no pain b)knee rom - 100 c) xray – uniting	a) no pain b) knee rom - 100 c) xray – uniting	a) no pain b) knee rom – 100 c) ankle rom full c) xray – united	17	No
23	28	M	4	Assault	AOC.1.2	a) Distal tibia locking plate 9 hole	a) no pain b) ankle rom 20(DF)/30(PF) c) xray - uniting	a) no pain b) ankle rom 20(DF)/40(PF) c) xray - uniting	a) no pain b) ankle rom 20(DF)/40(PF) c) xray – united	18	No
24	45	M	7	RTA	AO.A.1.1	a) hockey plate 11 hole b)3 CCS	a) no pain b) knee rom - 80 c) xray - uniting	a) no pain b) knee rom - 90 c) xray - uniting	a) no pain b) knee rom - 100 c) xray – united	21	No
25	30	M	3	RTA	AO.B.1.2	a) hockey plate 11 hole	a) no pain b) knee rom - 100 c) xray - uniting	a) no pain b) knee rom - 110 c) xray – uniting	a) no pain b) knee rom - 120 c) xray – united	19	No
26	52	F	6	RTA	AO.C.1.3	a) hockey plate 9 hole b)2 CCS	a) no pain b) knee rom - 90 c) xray – uniting	a) no pain b) knee rom - 90 c) xray - uniting	a) no pain b) knee rom - 100 c) xray – united	18	No
27	25	M	2	RTA	AO.B.1.2	a) hockey plate 9 hole	a) no pain b)knee rom - 100 c) xray - uniting	a) no pain b)knee rom - 120 c) xray – uniting	a) no pain b) knee rom - 130 c) xray – united	18	No
28	28	F	4	RTA	AO.A.1.3	a) hockey plate 11 hole b)2 CCS	a) no pain b) knee rom - 100 c) xray - uniting	a) no pain b) knee rom - 110 c) xray - uniting	a) no pain b) knee rom - 120 c) xray – united	15	No
29	45	M	5	RTA	A.O.A.1.2.	a) hockey plate 7 hole	a) no pain b) knee rom - 80 c) xray - uniting	a) no pain b) knee rom - 90 c) xray - uniting	a) no pain b)knee rom - 100 c) ankle rom full c) xray – united	18	No
30	60	F	7	Domestic fall	A.O.A.1.3	a) hockey plate 7 hole	a) no pain b)knee rom - 70 c) xray - uniting	a) no pain b) knee rom - 80 c) xray - uniting	a) no pain b) knee rom - 90 c) xray – united	19	No

**Table II:** Outcome classification based on SJLAM criteria^3^

Excellent	Range of movement of adjacent joints 80-100 % of normal. No pain in performing daily activities.
Good	Range of movement of adjacent joints 60 -80% normal. Pain not enough to cause any modification of patient daily routine.
Fair	Range of movement of adjacent joints 30–60% normal. Pain enough to cause restriction patients daily activities.
Poor	Range of movement of adjacent joints less than 30% of normal. Pain enough to cause severe disability or non union.

Clinical thinking has shifted from mechanical concept of absolute stability to the biologic concept of indirect reduction and relative stability using minimally invasive approach^12^. MIPO technique reduces the surgical trauma and maintains a biologically favorable environment for healing of the fracture^13^. However, minimally invasive techniques do not allow direct visualisation of the fracture, and hence intraoperative fluoroscopy is required to confirm the reduction^14^.

In our study, we favoured early surgical fixation. Majority of patients were operated within the first week of injury with the mean time to surgery being seven days. There is evidence in the literature which suggest that delayed intervention of these fractures make reduction more difficult^15^.

In our study, the average time of union was 17 weeks which was comparable to other studies on percutaneous plating of tibial fractures^16,17^. Complication rate was low. Our incidence of complications included one case of superficial infection and one case of non-union. Superficial infection healed by daily dressing under antibiotic cover. The cause of non- union was early weight bearing, comminution, fracture pattern, and was the first case in our learning curve. This case required a second surgery with bone grafting to achieve union.

In our study, all the fractures had good reduction and the location of plate was good.

The highlights of this study were absence of deep infection, high rate of union with average time of 17 weeks and early mobilization. The excellent success rate was achieved due to indirect or closed reduction of fracture without disturbing fracture hematoma. The limitations in our study were the small sample size and the lack of a control group. Another major pre-requisite was the surgical skill and experience required to carry out the procedure accurately.

## Consent

A written, informed consent was obtained from all the patients authorising the treatment, radiological and photographic documentation. They were informed and consented that the data would be submitted for publication.

## Conflict of Interest

Each author certified that he had no conflict of commercial interest in connection with the study. Each author certifies that his institution has approved the human protocol for this investigation and that all investigations were conducted in conformity with ethical principles of research, and informed consent by the patients for participation in the study was obtained.
